# Modelling Electron Channeling Contrast Intensity of Stacking Fault and Twin Boundary Using Crystal Thickness Effect

**DOI:** 10.3390/ma14071696

**Published:** 2021-03-30

**Authors:** Hana Kriaa, Antoine Guitton, Nabila Maloufi

**Affiliations:** 1 Arts et Métiers-LEM3, Université de Lorraine-CNRS, 7 rue Félix Savart, 57070 Metz, France; hana.kriaa@univ-lorraine.fr (H.K.); antoine.guitton@univ-lorraine.fr (A.G.); 2 Laboratory of Excellence on Design of Alloy Metals for Low-mAss Structures (DAMAS), Université de Lorraine, 57073 Metz, France

**Keywords:** dynamical theory of electron diffraction, ECCI, modelling BSE intensity, perfect and imperfect crystal, planar defects, twin boundary, stacking fault, crystal thickness

## Abstract

In a scanning electron microscope, the backscattered electron intensity modulations are at the origin of the contrast of like-Kikuchi bands and crystalline defects. The Electron Channeling Contrast Imaging (ECCI) technique is suited for defects characterization at a mesoscale with transmission electron microscopy-like resolution. In order to achieve a better comprehension of ECCI contrasts of twin-boundary and stacking fault, an original theoretical approach based on the dynamical diffraction theory is used. The calculated backscattered electron intensity is explicitly expressed as function of physical and practical parameters controlling the ECCI experiment. Our model allows, first, the study of the specimen thickness effect on the channeling contrast on a perfect crystal, and thus its effect on the formation of like-Kikuchi bands. Then, our theoretical approach is extended to an imperfect crystal containing a planar defect such as twin-boundary and stacking fault, clarifying the intensity oscillations observed in ECC micrographs.

## 1. Introduction

Microstructures and crystalline defects such as grain boundaries, dislocations, twins and stacking fault in materials have a fundamental and complex influence on their properties. For instance, dislocation interactions with grains boundaries and/or their nucleation under stress is one of the keys of the mechanical behavior of metals. Besides, having strong interactions with impurities in semiconductors, dislocations act as non-radiative recombination centers [1,2]. Moreover, planar defects such as native twins and/or deformation twinning have to be considered to enlighten the mechanisms of metals plastic deformation; strain accommodation [3,4]. For example, the CrMnFeCoNi high entropy alloy exhibit at room temperature deformation by dislocation slip whereas at very low temperature stacking faults and twinning act in addition [5]. Recent work has shown that the stacking faults role is predominant in response to loading in CoCrNiW metastable alloy [6]. On the other hand, the presence of such defects in materials for optoelectronic devices induces local strains leading to the degradation of their performances [7,8].

This is why, over the years, many characterization techniques and models were developed and adapted to better understand the role of these defects and thus optimize material properties using tailor-made elaboration and treatment procedures. Among these techniques, Electron Channeling Contrast Imaging (ECCI) is used in a Scanning Electron Microscope (SEM) for the observation and characterization of these defects in bulk materials [9,10,11]. ECCI is a non-destructive technique that provides Transmission Electron Microscope (TEM)-like diffraction contrast imaging of defects [10,12,13,14]. It is based on the electron channeling phenomenon, where electrons channel down the crystal planes for a given incidence angle between the incident beam and the crystallographic {hkl} planes. For such condition (channeling), the presence of crystalline defects modify locally the arrangement of the atomic columns and produce changes in the BackScattered Electron Intensity (I_BSE_), thus leading to a contrast.

In order to understand the origin of the Electron Channeling Contrasts (ECC), several theoretical approaches, based on the conventional dynamical diffraction theory, were developed. Some of them focused on the integration of the Howie–Whelan two-beam equations and used computational algorithms to generate the Electron Channeling Pattern (ECP) contrasts on a perfect crystal [15,16,17,18]. Others describe the electron beams, inside the crystal, by a superposition of Bloch waves, which are governed by different inelastic scattering process (single and multiple scattering process) [19,20,21,22,23].

Such theories model the observed contrast for the ECP and defects. Spencer et al. used the many-beam dynamical diffraction theory and the Bloch wave model [20]. They demonstrated that for a perfect crystal the I_BSE_ profiles of the bands forming the channeling pattern exhibit the main experimental features with a modulated intensity in the central region bounded by dark edges. In addition, in their theoretical model, the increase of the specimen thickness (above 1000 nm) produces an increase of the contrast and of the background intensity. More recently, Winkelmann obtained a theoretical channeling pattern corresponding to an experimental ECP using dynamical many-beam simulations based on the Bloch wave approach and on the forward–backward approximation [24].

The different theoretical approaches, cited above, were extended to the case of an imperfect crystal containing defects such as stacking faults [25] and dislocations [19,20]. For example, in the presence of a planar defect, a faulted plane separates two perfect crystals; for modelling, the upper crystal is held fixed while the lower one is translated by a vector **R** and/or rotated through an angle β about a vector **v** [26]. For example, these approaches showed that the calculated I_BSE_ profile of stacking fault exhibits damped fringes of depth periodicity ξ_g_ as observed experimentally [19,20,25]. Depending on the **g.R** sign (where **g** is the diffraction vector and **R** is the displacement vector of the fault) the contrast of the first fringe can be bright or dark. However, despite this important contribution to the theory of the channeling contrast of defects, the publications presenting these models did not display neither detailed calculations nor a usable analytical expression of the I_BSE_ leading to misunderstanding, and in most cases, a comparison between theoretical and experimental results is still missing [18,19,20,25].

In order to calculate the I_BSE_ around dislocations, Kriaa et al. developed a theoretical model based on the Bloch wave approach of the dynamical diffraction [13]. Their model leads to an explicit analytical formula of the BackScattered Electron (BSE) signal as function of various physical parameters controlling the ECCI experiment [12,13]. They demonstrated that screw and edge dislocations parallel to the sample surface have the same appearance of BSE contrast profiles for different diffraction conditions and they confirmed theoretically the use of the invisibility criteria in ECCI [13,27].

Nevertheless, despite these full experimental and theoretical studies devoted to dislocations and somewhat less to stacking faults, to our knowledge none explained the experimental ECC contrasts generated by twin-boundaries or attempted their modelling. Therefore, as for dislocations, we propose a theoretical approach for modelling the contrast intensity generated by twins in ECCI experiment.

The twinning is, in fact, a mechanism in which a region of a crystal undergoes a homogeneous shear that leads to a shift (and/or rotation) of a part of the crystal [26,28]. For coherent twin boundary, two visible crystals are separated by a planar surface intercepting the sample surface with an angle so that the crystal thickness varies from this surface. Therefore, the contrast obtained by a twin-boundary is equivalent to that produced by a wedge-shaped specimen (thin perfect crystal with a variable thickness). For this reason, further theoretical investigations concerning the influence of the crystal thickness on the BSE yield are necessary to understand the ECC of twins. For that we, first, developed a theoretical model resulting in an explicit formula of the BSE intensity for a perfect crystal versus its thickness (Section 2).

It is to be noted that a twin-boundary is a particular grain boundary where a fraction of lattice sites in one crystal is common to the other crystal. Therefore, some planes are common and give common reflections.

In the simple case of a coherent twin-boundary with a defined flat interface, it results from cumulative stacking faults (each of them shifting the crystal) on adjacent planes, with the difference that **R** is not constant moving away from the boundary plane (but constant along it), and thus **R** is not directly connected to the crystal lattice [26,28]. Hence, in the second part of this contribution, the strategy used to evaluate the BSE yields produced by a twin boundary is easily transposed to model the contrast generated by stacking faults (Section 3), taking advantage, in a simple way, from the explicit formula relating the BSE intensity of a perfect crystal to its thickness.

## 2. Materials and Methods

### Experimental Analysis of a Special Grain Boundary: Twin-Boundary

**Figure 1:**
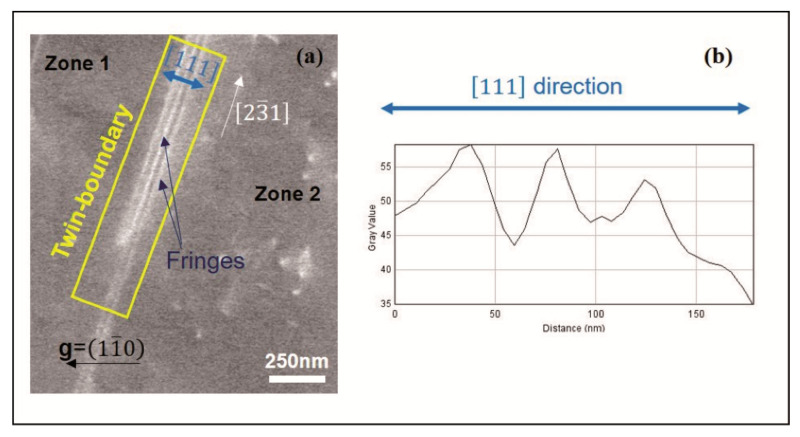
(**a**) Electron Channeling Contrasts (ECC) micrograph and (**b**) the experimental profile (obtained from the zone indicated by the blue arrow) of a true twin-boundary observed in TiAl for the diffraction condition **g** = (1$\bar{1}$0).

Figure 1a is an ECC micrograph of a twin-boundary already characterized by Electron BackScatter Diffraction (EBSD) [14]. It is carried out using a working distance of 7.3 mm, an acceleration voltage of 10 kV, and a pole piece mounted Backscattered Electron detector in a Zeiss Auriga electronic microscope (Zeiss SEM, Oberkochen, Germany). Briefly, the observed twin-boundary separates two grains (labeled zone 1 and zone 2) observed in the γ phase of a TiAl alloy for the diffraction condition **g** = (1$\bar{1}$0). The surface planes of zone 1 and zone 2 are, respectively, (457) and (013). The common direction on the surface to both grains is [2$\bar{3}$1]. The twin-boundary plane, which intercepts both (457) and (013) planes along direction [2$\bar{3}$1], is the (111) plane. This latter is inclined of about 11° relative to the surface of observation.

The contrast generated by this inclined planar defect starts with an intense bright line, in the [2$\bar{3}$1] direction, that marks its intersection with the surface. Next to this line, the contrast takes the form of bright and dark fringes, along the [111] direction, which indicates the intensity oscillations as shown in the experimental profile (indicated by the blue arrow in Figure 1a,b). The theoretical interpretations of this contrast are explained in the following section.

## 3. Theoretical Models

### 3.1. Contribution of a Thin Perfect Crystal to the BSE Signal

In order to calculate the contrast generated by a twin boundary or a stacking fault, we propose to study the modulation of the BSE signal in function of the specimen thickness t in a perfect crystal. To do so, we follow the Bloch wave approach presented in the textbook of Reimer [29] which gives the total BSE signal of a slice of a thickness dz situated at a depth z in the perfect crystal as following:

(1) $$\frac{d\eta }{\mathrm{dz}}{\;=\;N\sigma }_{B}{\;\{\psi \psi }^{*}+\;(1-\underset{j}{\sum }{\left|C_{0}{}^{\left(j\right)}\right|}^{2}e^{{[-4\pi q}^{\left(j\right)}z]})\}$$

where N is the atom number per unit of volume, σ_B_ is the backscattering cross-section through angles larger than 90° and ψ ψ* is the probability for the Bloch wave to be backscattered at a depth z. The last terms (in parentheses) in Equation (1) describes the electrons that are removed from the Bloch wave field by scattering before reaching the slice dz.

For that, the Equation (1) was integrated from z = 0 to z = t. This gives a new BSE coefficient ∆η’. The latter is presented as the sum of two terms:

(2) $$∆\eta^{\prime }(\omega ,\;t)\;=\;∆\eta (\omega )\;+\;T(\omega ,\;t)$$

Note that ∆ indicates that only the total I_BSE_ due to orientation contrast is calculated. Here, the atomic number and the surface inclination contributions are not considered.

Thus, ∆η is the BSE coefficient for a perfect crystal, independent of thickness t, obtained by the integration of Equation (1) on the total interaction depth from z equal zero to z tends towards infinity [29]:

(3) $$∆\eta \;=\;\frac{{N\sigma }_{B}}{4\pi }{\;\xi }_{0}^{\prime }(-\frac{\omega \;+\frac{\xi_{0}^{\prime }}{\xi_{g}^{\prime }}}{{1+(\omega )}^{2}-(\frac{\xi_{0}^{\prime }}{\xi_{g}^{\prime }}{)}^{2}}+\frac{\omega }{{1+(\omega )}^{2}{+[(1+(\omega )}^{2})(\frac{\xi_{0}^{\prime }}{\xi_{g}}{)]}^{2}})*$$

where ω the deviation parameter, $\xi \begin{array}{c} \prime \\ 0 \end{array}$ and $\xi \begin{array}{c} \prime \\ g \end{array}$ are the absorption lengths and ξ_g_ is the extinction distance. This Equation (3) corresponds to the intensity profile of an isolated pseudo-Kikuchi band [19,20,29]. Note that in the book of Reimer (Reimer, 1998), Equation (3) contains an error: It is written 2π to the denominator instead of 4π. 

T is also a BSE signal which is expressed in terms of ω and t. The limit of T when t tends towards infinity is zero.

It is important to note that in this model the column approximation is used [26]. It consists in dividing the sample into narrow columns (a few nanometers of wide) parallel to the direction of the incident wave (usually z-direction, normal to the sample surface). The generated intensity of a column depends only on the diffraction events that take place in the same column (no interaction with neighboring columns).

It should be mentioned also that for modelling the profiles Δη’ = f(ω), we will need the material-specific parameters: ξ_g_:$\xi \begin{array}{c} \prime \\ 0 \end{array}$ and $\xi \begin{array}{c} \prime \\ g \end{array}$. Such parameters are tabulated in the literature for a given acceleration voltage E and diffraction vector **g** for the following materials: Al, Si, Cu, Ge and Au [29]. 

In this paper we present the profiles calculated in the case of Al with such parameters: E = 20 kV, **g** = (220), ξ_g_ = 50 nm, $\xi \begin{array}{c} \prime \\ 0 \end{array}$ = 140 nm and $\xi \begin{array}{c} \prime \\ g \end{array}$ = 600 nm. Note that the same BSE intensity profile appearance are obtained for the other materials.

**Figure 2:**
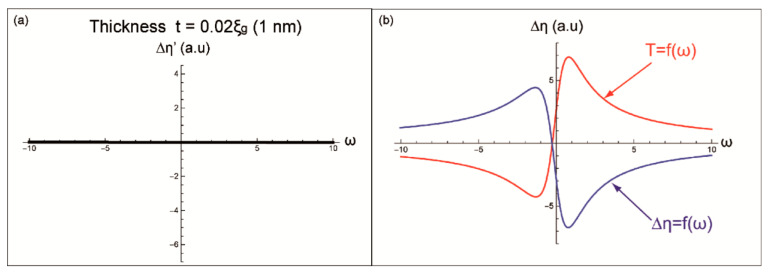
Modeled profiles of (**a**) Δη’(ω), (**b**) T(ω) and Δη(ω) for a thickness t = 0.02 ξ_g_ (1 nm).

**Figure 3:**
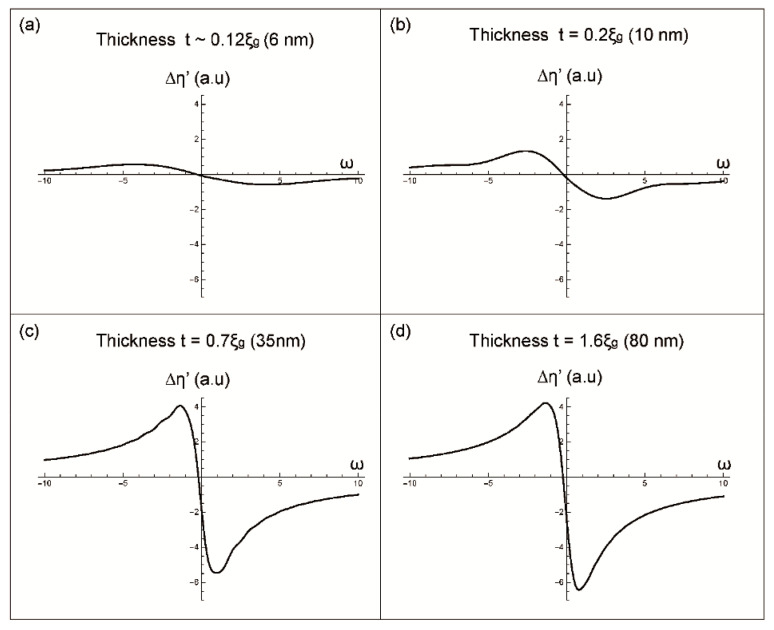
BSE intensity profiles of a pseudo-Kikuchi band generated for different sample thicknesses (**a**) t ≈ 0.12ξ_g_ (6 nm), (**b**) t = 0.2ξ_g_ (10 nm), (**c**) t = 0.7ξ_g_ (35 nm) and (**d**) t = 1.6ξ_g_ (80 nm).

The term T(ω,t) generates different profiles depending of the thickness. While Δη = f(ω) corresponds to a contribution to the BSE yields independent on this parameter. Therefore, different curves of Δη’(ω) will be generated according to the chosen t (Figure 2 and Figure 3).

- For a thin thickness t = 0.02ξ_g_ (~1 nm,) the curve of Δη’(ω) corresponds to a constant function close to 0 (Figure 2a). In Figure 2b, the red and blue curves represent, respectively, T(ω,t) and Δη(ω) for t = 0.02 ξ_g_.

It is interesting to know that in all calculated profiles the background noise level will be taken as a reference (at the zero level of the *y*-axis). So that any negative value will correspond to a BSE intensity lower than the background.

- For a thickness t= 0.12ξ_g_ (6 nm), the slight Δη’ variations are between −0.58 (a.u.) and 0.58 (a.u.) as it is shown in Figure 3a. Such variations are due to the slight contribution of the term T(ω).
- For a thickness t = 0.2ξ_g_ (10 nm), a larges peak (for negative values of ω) and a hollow (for positive values of ω) appear (see Figure 3b). In addition, it is noted that the more the thickness increases, the more the amplitude of Δη’(ω) increases. These same observations are accentuated for the following thicknesses.
- For the thicknesses t = 0.7ξ_g_ (35 nm) and t = ξ_g_ (50 nm), the two profiles have, almost, the same appearance: Peak and hollow less spread than those obtained for t = 0.2ξ_g_. In addition, oscillations on the sides of these curves appear (Figure 3c for t = 0.7ξ_g_).

Starting from a thickness t = 1.6ξ_g_ (80 nm), the appearance of the total Δη’ curve becomes identical to that of Δη (Figure 3d) corresponding to the contribution to BSE from an infinite or thick crystal. In this case, the contribution of the term T in the formation of the pseudo-Kikuchi band contrast becomes null (Δη prevails over since the limit of T when t tends towards infinity is zero).

In summary, the formation of the I_BSE_ profile for a perfect crystal occurs under different steps. Firstly, for thickness below 0.2ξ_g_, no band contrast is observed: For small thicknesses, the transmission of electrons takes place by different processes such as channeling, Bragg diffraction and inelastic scattering. This results in a low backscattered signal insufficient to form a Kikuchi band. Then, a transitory step is obtained in which oscillations are observed, on the sides of the curves, that disappear at t = 1.6ξ_g_. This latter thickness is, indeed, sufficient to generate an important backscattered signal sensitive to the orientation of the beam relative to the crystalline planes and thus to take advantage of the channeling phenomenon for characterizing defects. Therefore, the BSE intensity modulation allows the formation of the like-Kikuchi band with its main characteristics (dark line for channeling position (edge band), brighter band for θ < θ_c_). The text continues here.

### 3.2. Modelling the BSE Contrast Generated by a Coherent Twin Boundary

As already mentioned, the theoretical approach used to calculate the BSE signal produced by a twin boundary can be explored to model the contrast generated by stacking faults. In the case of a stacking fault (labeled SF in the following equations), two crystals are translated by a vector **R** and/or rotated through an angle β about a vector **v [26]**: Both crystals contribute to the contrast generation. For simplification reasons we will suppose that β = 0 and that the interface is plane, i.e., parallel to a crystallographic plane. The faulted plane is situated in a depth z_SF_. Here, we have to consider a new deviation parameter, for the slightly shifted crystal part:

ω + ω_SF_ where ω_SF_ = (**g.R**)ξ_g_ (4)

where ω is the deviation from the exact Bragg position in the perfect crystal below the stacking fault. The scalar product **g.R** represents the supplementary deviation ω_SF_ due to the displacement of the crystal below the fault plane relative to the crystal above. It is important to note that for the above crystal ω_SF_ is zero.

If we consider a column located at a position x for which corresponds a stacking fault depth z_SF_, the total generated BSE signal can be expressed as follow:

(5) $$\eta_{\mathrm{SF}}\left(\omega \right)=\int_{0}^{z_{\mathrm{SF}}}\frac{\partial \eta (\omega )}{\partial z}\mathrm{dz}+\int_{z_{\mathrm{SF}}}^{\infty }\frac{\partial \eta (\omega \prime )}{\partial z}\mathrm{dz}$$

So, the total BSE signal can be written as follow:

(6) $$\eta_{\mathrm{SF}}\left(\omega \right)=\int_{0}^{z_{\mathrm{SF}}}\frac{\partial \eta (\omega )}{\partial z}\mathrm{dz}+\int_{0}^{\infty }\frac{\partial \eta (\omega \prime )}{\partial z}\mathrm{dz}-\int_{0}^{z_{\mathrm{SF}}}\frac{\partial \eta (\omega \prime )}{\partial z}\mathrm{dz}$$

**Figure 4:**
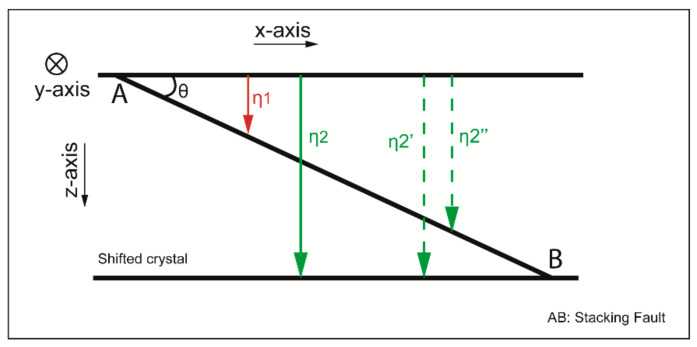
Explanatory schematic of the BSE signal generated from a crystal containing an inclined stacking fault. The red array represents the BSE signal generated from the perfect crystal above the fault plane. The BSE generated by the shifted crystal, η_2_, corresponds to a combination between the BSE signal represented with dotted green arrays.

To simplify:

(7) $$\eta_{\mathrm{SF}}\left(\omega \right)=\eta_{1}\left(\omega \right)+\eta_{2}^{\prime }\left(\;\omega \;\prime \;\right)-\eta_{2}^{\prime \prime }\left(\;\omega \;\prime \;\right)↔\eta_{\mathrm{SF}}\left(\omega \right)=\eta_{1}\left(\omega \right)+\eta_{2}\left(\omega \prime \right)$$

where η_1_ and η_2_ represent the BSE generated, respectively, from the perfect crystal above the stacking fault and from the shifted one as it is represented in the schematic of Figure 4: η_1_ and $\eta \begin{array}{c} \prime \prime \\ 2 \end{array}$ correspond to the contribution of a sample with z_SF_ thickness on the BSE signal, $\eta \begin{array}{c} \prime \\ 2 \end{array}$ represents the variation of this signal on a perfect crystal.

Considering that:

(8) $$z_{\mathrm{SF}}=x\;\tan(\theta )$$

The variation of the I_BSE_ (experimentally: Intensity profile collected in the direction perpendicular to the fringes) is then obtained as a function of x; distance away from the intersection of the defect with the surface (θ is the angle between the stacking fault plane and the surface).

#### 3.2.1. For **g.R** ≠ 0

**Figure 5:**
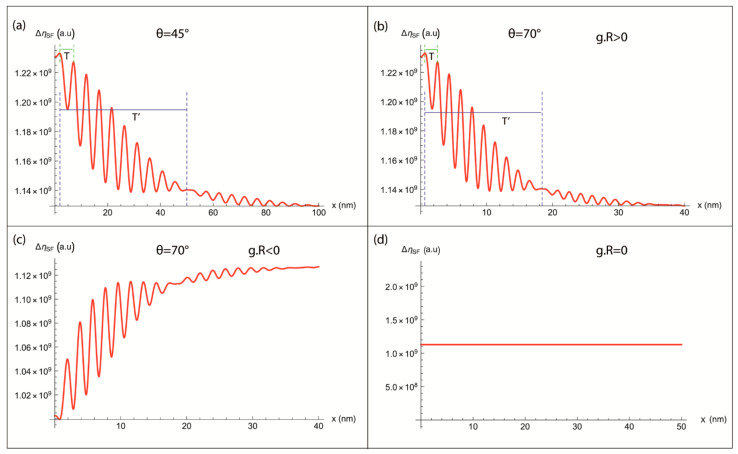
BackScattered Electron Intensity (I_BSE_) profile calculated as a function of x, distance away of the intersection of the defect with the surface, for (**a**) **g.R** ≠ 0 and θ = 45°, (**b**) **g.R** > 0, θ = 70° and (**c**) **g.R** < 0, θ = 70° and (**d**) **g.R** = 0. The green and blue lines indicate, respectively, the spatial periods T and T’.

The calculated BSE signal as a function of x, distance away of the intersection of the defect with the surface, is represented in Figure 5 for **g.R** ≠ 0 (where **g** = (220)). The curves, in Figure 5a,b, show that the inclined stacking fault starts with an intense light line at the intersection of the defect with the surface (x = 0 nm). For increasing x values, the calculated contrast fades with oscillations in agreement with experimental ECC images of twin boundary (Figure 1) and stacking fault (Figure 12 in [6]). Furthermore, the more the inclination angle θ is important the more the spatial periods T and T’ (indicated, respectively, by the green and the blue lines in Figure 5a,b) become lower (T ≈ 5 nm, T’ ≈ 50 nm for θ = 45°, and T ≈ 2 nm, T’ ≈ 18 nm for θ = 70°), and the more the contrast fades quickly. Moreover, the modeled profiles start with an intensity peak (see Figure 5b) and hollow (see Figure 5c), respectively, for **g.R** > 0 and for **g.R** < 0. 

#### 3.2.2. For **g.R** = 0

The theoretical intensity profile, generated form an inclined stacking fault, calculated from our model in the case of **g.R** = 0 is represented in Figure 5d. This latter shows a line parallel to the *x*-axis which corresponds to the background signal; the defect is then invisible. It is important to note that experimentally such defect can be invisible even out of these invisibility conditions. For example, for a stacking fault parallel to the surface of the specimen, **g.R** = 0 for all values of **g** lying in the faulted plane.

## 4. Conclusions

This paper presents an original theoretical model based on the Bloch wave approach of the dynamical diffraction theory for modelling the BSE signal as a function of the physical parameters governing an ECCI experiment. It results in an explicit formula of the variation of the I_BSE_ as a function of the thickness of the crystal aiming a twofold purpose: First, the formation of like-Kikuchi bands, and second the modelling of the contrast generated by a planar defect such as a coherent twin-boundary or a stacking fault. Considering a perfect crystal, we demonstrate that the formation of the I_BSE_ profile of a like-Kikuchi band requires a sufficient thickness depending on the extinction distance ξ_g_; at least 1.6ξ_g_ in the case of Al. Under such conditions, the sensitivity of the BSE yield BSE is adequate, and the channeling phenomenon can be used to determine crystal orientation or to contrast defects.

In order to understand the I_BSE_ generated by a planar defect, which in some cases displays fringes, our model was extended in an elegant and simple way to the case of an imperfect crystal containing a stacking fault or a coherent twin boundary inclined relative to the surface specimen. For these cases, the calculated BSE profiles exhibit an oscillatory regime: The amplitude of the oscillations decreases with the increasing values of the distance away from the intersection defect-surface due to the Bloch wave absorption. The oscillation frequency depends on different parameters such as the inclination angle θ and the extinction distance ξ_g_ in agreement with experimental observation. Furthermore, the inversion of the **g.R** sign led to the inversion of the planar defect contrast and no contrast is generated for **g.R** = 0 exactly as for dislocations.

## Data Availability

Data are contained within the article.
